# Incidence of bifid uvula and its relationship to submucous cleft palate and a family history of oral cleft in the Brazilian population^☆^

**DOI:** 10.1016/j.bjorl.2017.08.004

**Published:** 2017-08-24

**Authors:** Sizina Aguiar G. Sales, Maria Luiza Santos, Renato Assis Machado, Verônica Oliveira Dias, Jairo Evangelista Nascimento, Mario Sérgio Oliveira Swerts, Hercílio Martelli Júnior, Daniella Reis Barbosa Martelli

**Affiliations:** a Universidade Estadual de Montes Claros (Unimontes), Faculdade de Medicina, Montes Claros, MG, Brazil; b Universidade Estadual de Montes Claros (Unimontes), Programa de Pós-graduação em Cuidado Primário em Saúde, Montes Claros, MG, Brazil; c Universidade Estadual de Montes Claros (Unimontes), Faculdade de Odontologia, Montes Claros, MG, Brazil; d Universidade Estadual de Campinas (Unicamp), Faculdade de Odontologia de Piracicaba, Departamento de Diagnóstico Oral, Piracicaba, SP, Brazil; e Universidade de José Rosário Vellano, Faculdade de Odontologia, Centro de Reabilitação de Anomalias Craniofaciais, Alfenas, MG, Brazil

**Keywords:** Bifid uvula, Submucosal cleft palate, Cleft lip, Cleft palate, Children, Úvula bífida, Fissura palatina submucosa, Fissura labial, Fissura palatina, Crianças

## Abstract

**Introduction:**

Bifid uvula is a frequently observed anomaly in the general population and can be regarded as a marker for submucous cleft palate.

**Objective:**

In this study aimed to determine the frequency of bifid uvula and submucous cleft palate and their relationship with oral clefts in a Brazilian population.

**Methods:**

We conducted a transversal, descriptive and quantitative study of 1206 children between August 2014 and December 2015. A clinical examination of the children was conducted by means of inspection of the oral cavity with the aid of a tongue depressor and directed light. After the clinical examination in children, parents answered a questionnaire with questions about basic demographic information and their family history of oral clefts in their first-degree relatives. After application of the questionnaires, the information collected was archived in a database and analyzed by the statistical program SPSS^®^ version 19.0, by applying Chi-Square tests. Values with *p* < 0.05 were considered statistically significant.

**Results:**

Of the 1206 children included in this study, 608 (50.40%) were female and 598 (49.60%) were male (*p* = 0.773). The average age of children was 3.75 years (standard deviation ± 3.78 years). Of the 1206 children studied, 6 (0.5%) presented with bifid uvula. Submucosal cleft palate was not found in any child. When the family histories of children were examined for the presence of nonsyndromic cleft lip and/or cleft palate, no first degree relatives presented with the congenital anomaly.

**Conclusion:**

This study revealed that the incidence of bifid uvula and submucous cleft palate in this population was quite similar to previously reported incidence rates. Our study suggests an intensification of new reviews, with broader and diverse populations, seeking to associate the occurrence of bifid uvula, submucous cleft palate and oral clefts.

## Resumo

**Introdução:**

A úvula bífida é uma anomalia frequentemente observada na população em geral e pode ser considerada como um marcador de fissura palatina submucosa.

**Objetivo:**

Determinar a frequência de úvula bífida e fissura palatina submucosa e sua relação com fissura orais em uma população brasileira.

**Método:**

Realizamos um estudo transversal, descritivo e quantitativo de 1.206 crianças entre agosto de 2014 e dezembro de 2015. O exame clínico das crianças foi realizado por meio da inspeção da cavidade oral com auxílio de um abaixador de língua e luz direcionada. Após o exame clínico nas crianças, os pais responderam a um questionário com perguntas sobre informações demográficas básicas e antecedentes de fendas orais em familiares de primeiro grau. As informações coletadas foram arquivadas em um banco de dados e analisadas pelo programa estatístico SPSS^®^ versão 19.0, aplicando testes de Qui-Quadrado. Os valores com *p* < 0,05 foram considerados estatisticamente significativos.

**Resultados:**

Das 1.206 crianças incluídas neste estudo, 608 (50,40%) eram do gênero feminino e 598 (49,60%) do masculino (*p* = 0,773). A idade média das crianças foi de 3,75 anos (desvio-padrão ± 3,78 anos). Das 1.206 crianças estudadas, seis (0,5%) apresentavam úvula bífida. A fissura palatina submucosa não foi encontrada em nenhuma criança. Quando as histórias familiares de crianças foram examinadas quanto à presença de fissura de lábio e/ou palato não sindrômica, nenhum parente de primeiro grau apresentava esta anomalia congênita.

**Conclusão:**

Este estudo revelou que a incidência de úvula bífida e fissura palatina submucosa nesta população é bastante semelhante às taxas de incidência previamente relatadas. Nosso estudo sugere uma intensificação de novas revisões, com populações mais amplas e diversas, buscando associar a ocorrência de úvula bífida, fissura palatina submucosa e fissura orais.

## Introduction

Bifid uvula is a frequently observed anomaly in the general population.^1^ Its incidence varies according to racial groups.^2^ The incidence is higher in Indians and Mongols, average in Caucasians and less frequent in blacks.^2^, ^3^ Bifid uvula is often regarded as a marker for submucous cleft palate although this relationship has not been fully confirmed.^1^, ^4^ The bifid uvula has thus served as a tool for clinicians to detect the earliest signs of oral cleft.^4^

Submucous cleft palate is a congenital malformation with specific clinical features that were first described by Calnan and are known as “Calnan's triad”.^5^ The diagnostic signs of Calnan's triad are bifid uvula, midline soft palate muscle separation with an intact mucosal surface, and a midline posterior bony palate-notching defect.^6^ It has an estimated prevalence of 1:1250–1:5000.^7^ The OMIM database of Mendelian disorders lists submucous cleft palate as a clinical finding in approximately 40 distinct syndromes. Yet, in approximately 70% of cases, submucous cleft palate is an isolated finding.^8^

Nonsyndromic cleft lip and/or cleft palate (NSCL/P, OMIM # 119530) is the most common orofacial birth defect, occurring in 1 in 500–2500 live births worldwide.^9^ In Brazil, the prevalence varies from 0.36 and 1.54 per 1000 live births.^10^, ^11^ NSCL/P is caused by a complex interplay between environmental exposures and genetic and epigenetic factors. Although in the past decade multiple genetic variants have been associated with oral clefts, providing valuable insights into its genetic etiology, the disease-susceptibility genes identified so far only account for a small percentage of cases.^9^, ^12^

Therefore, the aim of the current study was to determine the frequency of bifid uvula and submucous cleft palate and their relationship with oral clefts in a Brazilian population.

## Methods

After proper approval of the Ethics Committee (no. 957.462), Institutional Review Board, we conducted a transversal, descriptive and quantitative study of 1206 children between August 2014 and December 2015. The children were assessed in primary or ambulatorial units of health. All units are Public Health Network Brazilian (Unified Health System). All of the study subjects were born in the same region of the Minas Gerais State, Brazil, and had similar social conditions.

A clinical examination of the children was conducted by means of inspection of the oral cavity with the aid of a tongue depressor and directed light. The use of light through the lantern allowed a direct view in front of the examiner (SAGS). The examination of the oral cavity aimed to verify the presence of a bifid uvula or submucosal cleft palate.

After the clinical examination in children, parents answered a questionnaire with questions about basic demographic information and their family history of oral clefts in their first-degree relatives (mother, father, son, daughter, and siblings).^13^ No parent declined to respond the questionnaire. The questionnaires were applied in a single session, always after the clinical examination of children. Children with congenital anomalies or syndromes were excluded from the study. This was initially performed as a pilot study.

The oral clefts were categorized, when present, into the following three groups, with the incisive foramen as a reference: 1) cleft lip (CL): includes complete or incomplete pre-foramen clefts, either unilateral or bilateral; 2) cleft lip and palate (CLP): includes unilateral or bilateral transforamen clefts and pre- or post-foramen clefts; and 3) cleft palate (CP): includes all post-foramen clefts, complete, or incomplete.^14^

After application of the questionnaires, the information collected were archived in a database and analyzed by the statistical program SPSS^®^ version 19.0, by applying Chi-square tests. Values with *p* < 0.05 were considered statistically significant.

## Results

Of the 1206 children included in this study, 608 (50.40%) were female and 598 (49.60%) were male (*p* = 0.773). The average age was 3.75 years (SD ± 3.78 years). There was a prevalence of non-Caucasians (764–63.3%) versus Caucasians (442–36.7%).

Of the 1206 children studied, 6 (0.5%) presented with bifid uvula. Submucosal cleft palate was not found in any child. When the family histories of children were examined for the presence of NSCL/P, no first-degree relatives presented with the congenital anomaly.

## Discussion and conclusion

The ancestry of individual inhabitants of the Minas Gerais state with oral clefts had been previously investigated.^15^, ^16^ The average ancestry contributions to patients with oral clefts were estimated as 87.5% European, 10.7% African, and 1.8% Amerindian.^15^

The term bifid uvula means the partial or full bifurcation of the uvula. The occurrence of bifid uvula has aroused interest because of the possibility of being considered a mild form of cleft palate or being associated with submucosal cleft palate.^4^, ^17^ Discovering bifidity of the uvula, however, may not be as simple as it first appears. Mucous viscosity can hold a notched or grossly bifid uvula together, making bifidity quite difficult to identify by routine oropharyngeal exam. Mucous viscosity can also prevent the identification of these anomalies intraoperatively, even after careful inspection and palpation.^4^

Bifid uvula is apparent in 0.44%–3.3% of normal individuals.^1^, ^18^ Of 1206 children examined in the present study, 6 (0.49%) presented with bifid uvula. As several studies^19^, ^20^, ^21^ showed a higher incidence of cleft palate in females, it is possible to assume a higher prevalence of bifid uvula in females as well. However, in our study of 6 cases of bifid uvula found, most occurred in males (5 vs. 1). Studies conducted by our group in the same State (Minas Gerais, Brazil), showed a predominance of cleft palate in females.^21^, ^22^ There are also other studies^18^, ^23^, ^24^ that have shown a higher occurrence of uvula bifida in males in agreement with our study.

Similar to other cases of cleft palate, submucosal cleft palate shows malpositioning of the palate muscles and may result in velopharyngeal insufficiency and hypernasality.^6^ However, submucosal cleft palate is more difficult to diagnose than other cases of cleft palate, in part because the soft and hard palates show no gap and only the uvula is bifid.^6^ This is in accordance with previously reported results that submucosal cleft palate is often diagnosed late.^25^, ^26^ One reason for late diagnosis may be a lack of alertness for obvious anatomical features of an underlying invisible cleft of the palate.^25^, ^26^ During intra-oral examination, more than 90% of the patients showed a bifid uvula, which was associated with submucosal cleft palate. This visual anatomical variation, however, remained undetected during screening of newborns after birth.^27^ Although, the presence of bifid uvula is constant for the occurrence of submucous cleft palate, in our study, of 1206 children evaluated, no cases of submucous cleft palate were found.

Although there has been marked progress in identifying the environmental and genetic risk factors associated with oral clefts, its etiology in most cases remains unclear.^9^ Studies have sought to correlate several changes with oral clefts.^28^ The occurrence of malignant neoplasms in relatives of patients with oral clefts^13^, ^28^ and dental anomalies^29^ has been reported in patients with oral clefts. In the present study, we could not identify any cases of oral clefts in relatives of children with bifid uvula.

Although children with bifid uvula may have changes in speech, hearing and swallowing, of the 6 children with bifid uvula uncovered in our study, these changes were not observed. All of their parents were told of the presence of the uvula bifida in their children. The cooperation of doctors such as pediatricians and otorhinolaryngologists who are in contact with several infants and young children, will be vital for the identification of bifid uvula. Children in whom bifid uvula is evident upon oral examination during regular health checkups should be examined by a specialist.^6^ Here, the important interactions between various health professionals, including doctors and dentists, are clearly visible.

In summary, this study revealed that 0.5% of patients with oral cleft showed bifid uvula in a Brazilian population. No patient presented submucous cleft palate and no first degree relatives had congenital anomaly. Our study suggests that an intensification of new reviews, with broader and diverse populations, seeking to associate the occurrence of bifid uvula, submucous cleft palate and oral clefts, is needed.

## Conflicts of interest

The authors declare no conflicts of interest.
